# Supplementary material for: Systematic review of the best evidence for resistance exercise in maintenance hemodialysis patients
Source: PLoS One. 2024 Dec 30;19(12):e0309798. doi: 10.1371/journal.pone.0309798 (PMC11684604; doi:10.1371/journal.pone.0309798)
Supplement: S2 Checklist — (DOCX) [file pone.0309798.s002.docx]

**Identification of studies via databases and registers**

Records removed *before screening*:

Duplicate records removed (n =80 )

Records marked as ineligible by automation tools (n = 0)

Records removed for other reasons (n =0 )

Records identified from*:

Databases (n =467 )

Registers (n = 0)

**Identification**

Records screened

(n =387 )

Records excluded**

(n = 206)

Reports sought for retrieval

(n =181 )

Reports not retrieved

(n =0 )

**Screening**

Reports assessed for eligibility

(n =181)

Reports excluded:

Not relevant outcomes or not trail (n = 65)

Inability to present useful data (n = 6)

Not exportable(n = 6)

Reports of included studies

(n = 16)

**Included**

*Consider, if feasible to do so, reporting the number of records identified from each database or register searched (rather than the total number across all databases/registers).

**If automation tools were used, indicate how many records were excluded by a human and how many were excluded by automation tools.

*From:*  Page MJ, McKenzie JE, Bossuyt PM, Boutron I, Hoffmann TC, Mulrow CD, et al. The PRISMA 2020 statement: an updated guideline for reporting systematic reviews. BMJ 2021;372:n71. doi: 10.1136/bmj.n71

For more information, visit: <http://www.prisma-statement.org/>
