# Supplementary figures and images for: Systematic review of the best evidence for resistance exercise in maintenance hemodialysis patients
Source: PLoS One. 2024 Dec 30;19(12):e0309798. doi: 10.1371/journal.pone.0309798 (PMC11684604; doi:10.1371/journal.pone.0309798)

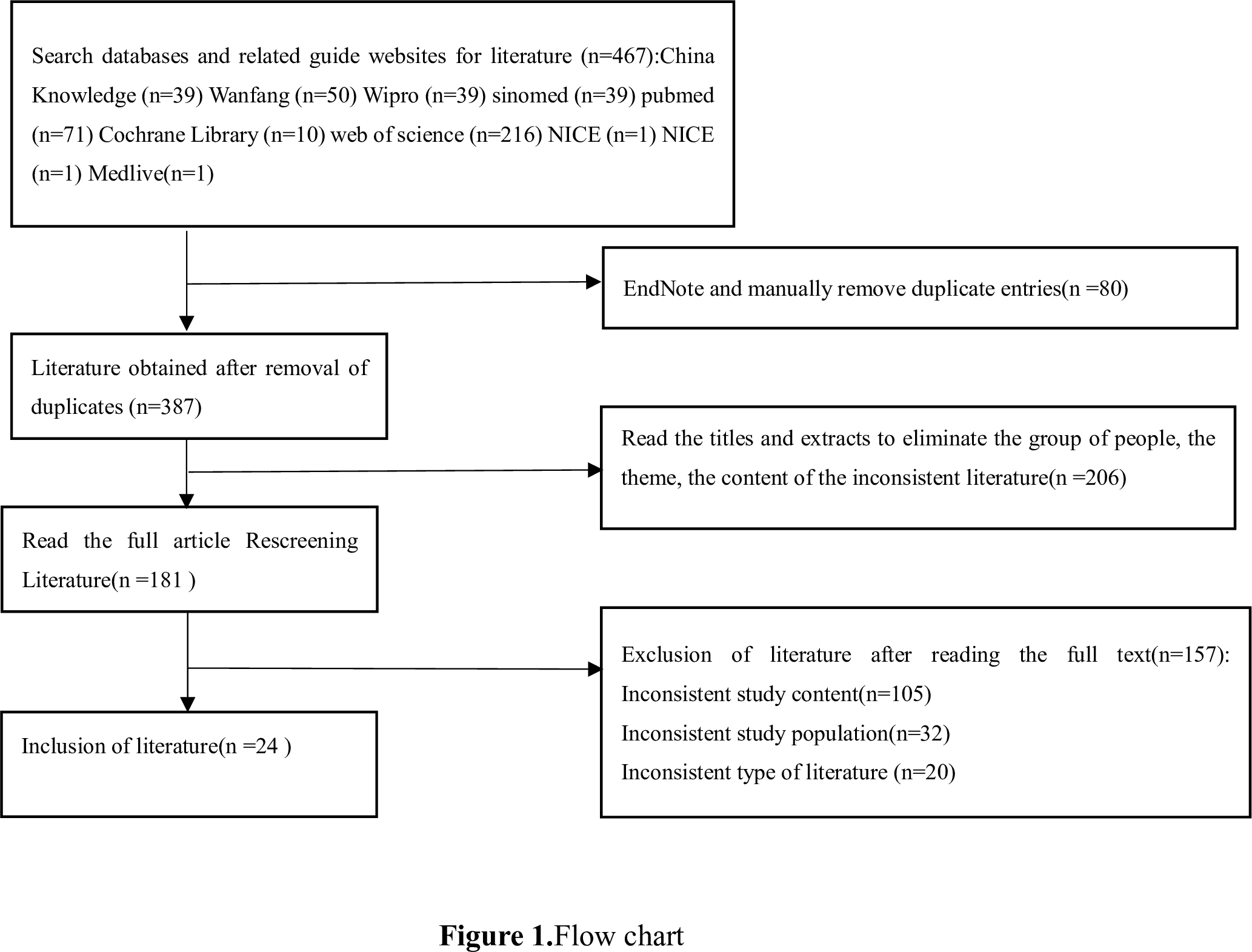

Supplement: S1 Fig — (DOCX) [file pone.0309798.s003.docx]
