# Supplementary material for: Systematic review of the best evidence for resistance exercise in maintenance hemodialysis patients
Source: PLoS One. 2024 Dec 30;19(12):e0309798. doi: 10.1371/journal.pone.0309798 (PMC11684604; doi:10.1371/journal.pone.0309798)
Supplement: S1 Table — (DOCX) [file pone.0309798.s004.docx]

**S1 Table. Quality Assessment Results of Guidelines**

The quality assessment for guidelines will be conducted using the Appraisal of Guidelines for Research and Evaluation (AGREE II) system.

| Literature | Quality assessment results |
| --- | --- |
| JBI Evidence Pre-grading and Evidence Recommendation Level System (2014 edition)[21] | A |
| Summary of evidence for resistance training in patients with breast cancer-associated lymphedema [22] | A |
| Renal Association Clinical Practice Guideline on Haemodialysis [23] | A |
| Clinical practice guideline exercise and lifestyle in chronic kidney disease[24] | A |

**Note** :The AGREE II evaluation system consists of six domains (Scope and Purpose, Participants, Rigour, Clarity, Application, and Independence), 23 main entries, and two overall evaluation entries. Each entry and the two overall assessment entries are graded on a 7-point scale, with 1 being ‘strongly disagree’, 7 being ‘strongly agree’, and the rest being graded from 2 to 6, depending on the circumstances. Therefore, the overall rating is A.
