# Supplementary material for: Systematic review of the best evidence for resistance exercise in maintenance hemodialysis patients
Source: PLoS One. 2024 Dec 30;19(12):e0309798. doi: 10.1371/journal.pone.0309798 (PMC11684604; doi:10.1371/journal.pone.0309798)
Supplement: S2 Table — (DOCX) [file pone.0309798.s005.docx]

**S2 Table. Quality Assessment Results of Expert Consensus**

Expert consensus will be evaluated according to the criteria established by the Joanna Briggs Institute (JBI) for Evidence-Based Healthcare (2016).

| Item  Literature | 1 | 2 | 3 | 4 | 5 | 6 | 7 |
| --- | --- | --- | --- | --- | --- | --- | --- |
| Expert consensus on the construction of renal rehabilitation system in hemodialysis room (center).[6] | yes | unclear | yes | yes | yes | unclear | yes |
| The BASES expert statement on exercise therapy for people with chronic kidney disease[25] | yes | yes | yes | yes | yes | unclear | yes |
| Expert consensus on exercise rehabilitation of adult patients with chronic kidney disease in China[26] | yes | yes | yes | yes | yes | unclear | yes |

**Note**:1. Are the sources of the ideas clearly labelled? 2. Whether the author is influential in the field? 3. Whether the views presented are centred on the interests of patients? 4. Whether the logical or empirical basis for the viewpoint is articulated? 5. Whether the analysis of the views is well-founded? 6. Are there any inconsistencies between the ideas presented and previous literature? 7. Whether the ideas presented are recognised by peers in the field? Evaluation results are expressed as yes, no, or unclear.
