# Supplementary material for: Systematic review of the best evidence for resistance exercise in maintenance hemodialysis patients
Source: PLoS One. 2024 Dec 30;19(12):e0309798. doi: 10.1371/journal.pone.0309798 (PMC11684604; doi:10.1371/journal.pone.0309798)
Supplement: S3 Table — (DOCX) [file pone.0309798.s006.docx]

**S3 Table. Quality Assessment Results of Systematic Reviews**

| Item  Literature | 1 | 2 | 3 | 4 | 5 | 6 | 7 | 8 | 9 | 10 | 11 | 12 | 13 | 14 | 15 | 16 |
| --- | --- | --- | --- | --- | --- | --- | --- | --- | --- | --- | --- | --- | --- | --- | --- | --- |
| Effects of Exercise on Muscle Fitness in Dialysis Patients: A Systematic Review and Meta-Analysis[9] | yes | yes | yes | yes | yes | yes | yes | yes | yes | yes | yes | yes | yes | yes | yes | yes |
| Intradialytic exercise training modalities on physical functioning and health-related quality of life in patients undergoing maintenance hemodialysis: systematic review and meta-analysis [27] | yes | yes | yes | yes | yes | yes | yes | yes | yes | yes | yes | yes | yes | yes | no | yes |
| Combined training is the most effective training modality to improve aerobic capacity and blood pressure control in people requiring haemodialysis for end-stage renal disease: systematic review and network meta-analysis.[28] | yes | yes | yes | yes | yes | yes | yes | yes | partial yes | yes | yes | yes | yes | yes | partial yes | yes |
| Effects of intradialytic exercise on cardiopulmonary capacity in chronic kidney disease: systematic review and meta-analysis of randomized clinical trials[29] | yes | yes | yes | yes | yes | yes | yes | yes | yes | no | yes | yes | yes | yes | yes | yes |
| Mesh meta-analysis of different exercise methods to improve walking ability in maintenance hemodialysis patients[30] | yes | yes | yes | yes | yes | yes | yes | yes | yes | no | yes | yes | yes | yes | yes | no |
| A systematic review and meta-analysis: Vinegar consumption on glycaemic control in adults with type 2 diabetes mellitus[31] | yes | yes | yes | yes | yes | yes | yes | yes | yes | yes | yes | yes | yes | yes | yes | yes |
| Exercise Interventions for Improving Objective Physical Function in End-Stage Kidney Disease Patients on Dialysis: A Systematic Review and Meta-Analysis[32] | yes | yes | yes | yes | yes | yes | yes | yes | yes | yes | yes | yes | yes | yes | yes | yes |
| Intradialytic Exercise in Hemodialysis Patients: A Systematic Review and Meta-Analysis. American journal of nephrology[33] | yes | yes | yes | yes | yes | yes | yes | yes | yes | yes | yes | yes | yes | yes | yes | yes |
| Exercise interventions on patients with end-stage renal disease: a systematic review[34] | yes | yes | yes | yes | yes | yes | yes | yes | yes | yes | yes | yes | yes | yes | yes | yes |

Systematic reviews will undergo quality assessment using the Assessment of Multiple Systematic Reviews 2 (AMSTAR 2).

**Note**: 1. Did the research questions and inclusion criteria for the review include the components of PICO? 2. Did the report of the review contain an explicit statement that the review methods were established prior to the conduct of the review and did the report justify any significant deviations from the protocol? 3. Did the review authors explain their selection of the study designs for inclusion in the review? 4. Did the review authors use a comprehensive literature search strategy? 5. Did the review authors perform study selection in duplicate? 6. Did the review authors perform data extraction in duplicate? 7. Did the review authors provide a list of excluded studies and justify the exclusions? 8. Did the review authors describe the included studies in adequate detail? 9. Did the review authors use a satisfactory technique for assessing the risk of bias (RoB) in individual studies that were included in the review? 10. Did the review authors report on the sources of funding for the studies included in the review? 11. If meta-analysis was performed did the review authors use appropriate methods for statistical combination of results? 12. If meta-analysis was performed, did the review authors assess the potential impact of RoB in individual studies on the results of the meta-analysis or other evidence synthesis? 13. Did the review authors account for RoB in individual studies when interpreting/ discussing the results of the review? 14. Did the review authors provide a satisfactory explanation for, and discussion of, any heterogeneity observed in the results of the review? 15. If they performed quantitative synthesis did the review authors carry out an adequate investigation of publication bias (small study bias) and discuss its likely impact on the results of the review? 16. Did the review authors report any potential sources of conflict of interest, including any funding they received for conducting the review? AMSTAR 2 consists of 16 entries, of which 7 are critical areas and 9 are non-critical areas. The scoring rules for AMSTAR 2 are strict, allowing only ‘yes’ or ‘no’ responses; if there are one or more ‘no’ responses in the critical areas, the overall quality of the systematic evaluation is rated as ‘low’; if there are two or more ‘no’ responses in the critical areas, the overall quality is rated as ‘low’. If one or more of the key areas are answered ‘no,’ the overall quality of the systematic evaluation is rated as ‘low’; if two or more of the key areas are answered ‘no,’ the overall quality is rated as ‘Very low’.
