# Supplementary material for: Systematic review of the best evidence for resistance exercise in maintenance hemodialysis patients
Source: PLoS One. 2024 Dec 30;19(12):e0309798. doi: 10.1371/journal.pone.0309798 (PMC11684604; doi:10.1371/journal.pone.0309798)
Supplement: S4 Table — (DOCX) [file pone.0309798.s007.docx]

**S4 Table. Quality Assessment Results of Randomized Controlled Trials**

Quality assessment for randomized controlled trials will adhere to the Joanna Briggs Institute's Randomized Controlled Trial Assessment Criteria (2016)

| Item  Literature | 1 | 2 | 3 | 4 | 5 | 6 | 7 | 8 | 9 | 10 |
| --- | --- | --- | --- | --- | --- | --- | --- | --- | --- | --- |
| Effect of intradialytic progressive resistance exercise on physical fitness and quality of life in maintenance haemodialysis patients[8] | yes | yes | yes | yes | unclear | yes | yes | yes | yes | yes |
| Resistance training improves sleep quality, redox balance and inflammatory profile in maintenance hemodialysis patients: a randomized controlled trial[12] | yes | yes | yes | not applicable | not applicable | yes | yes | yes | yes | Yes |
| Effects of intradialytic resistance exercise on systemic inflammation in maintenance hemodialysis patients with sarcopenia: a randomized controlled trial[35] | yes | yes | yes | unclear | unclear | yes | yes | yes | yes | Yes |
| Combined aerobic resistance exercise improves dialysis adequacy and quality of life in patients on maintenance hemodialysis[36] | yes | yes | yes | unclear | unclear | yes | yes | yes | yes | Yes |
| Effect of progressive resistance exercise on exercise ability, nutrition index and sleep quality in maintenance hemodialysis patients[37] | yes | yes | yes | no | no | yes | yes | yes | yes | Yes |
| Progressive exercise for anabolism in kidney disease (PEAK): a randomized, controlled trial of resistance training during hemodialysis[38] | yes | yes | yes | unclear | unclear | yes | yes | yes | yes | Yes |
| Effect of Exercise Performed during Hemodialysis: Strength versus Aerobic[39] | yes | yes | yes | yes | unclear | Yes | yes | yes | Yes | Yes |
| Effect of Peripheral and Respiratory Muscle Training on the Functional Capacity of Hemodialysis Patients[40] | yes | yes | yes | not applicable | not applicable | Yes | Yes | Yes | yes | Yes |
| Effect of Chair Stand Exercise on Activity of Daily Living: A Randomized Controlled Trial in Hemodialysis Patients[41] | yes | yes | yes | no | no | yes | Yes | Yes | yes | yes |
| Effects of Resistance Exercise Training and Nandrolone Decanoate on Body Composition and Muscle Function among Patients Who Receive Hemodialysis[42] | yes | yes | yes | no | no | yes | yes | yes | yes | Yes |

**Note**: 1.Whether or not a truly randomised grouping method is used? 2. Whether or not the study subjects were blinded? 3. Whether or not allocation hiding is applied to the grouping? 4. Whether or not the outcomes of lost subjects were described and included in the analyses? 5. Whether or not outcome assessors are blinded? 6. Whether the experimental and control groups were comparable at baseline time? 7. Whether the groups received the same interventions other than the one to be validated? 8. Whether outcome indicators were measured in the same way for each group of study participants? 9. Credibility of outcome measures? 10. Appropriateness of the method of analysing the information? The JBI Quality Assessment Tool on RCTs consists of 13 entries that evaluate the overall quality of RCTs in terms of randomisation, control, implementation of blinding, follow-up of study participants, and collection and analysis of outcome metrics during the course of the study, each of which is adjudicated using a Yes, No, Unclear, and Not Applicable.
